# Editorial: Modulation of NMDA Receptors: From Bench Side to Clinical Applications in Psychiatry

**DOI:** 10.3389/fpsyt.2022.896327

**Published:** 2022-04-13

**Authors:** Nataša Petronijević, Hsien-Yuan Lane, Nevena V. Radonjić

**Affiliations:** ^1^Faculty of Medicine, Institute of Medical and Clinical Biochemistry, University of Belgrade, Belgrade, Serbia; ^2^Graduate Institute of Biomedical Sciences, China Medical University, Taichung, Taiwan; ^3^Department of Psychiatry & Brain Disease Research Center, China Medical University Hospital, Taichung, Taiwan; ^4^Department of Psychology, College of Medical and Health Sciences, Asia University, Taichung, Taiwan; ^5^Department of Psychiatry and Behavioral Sciences, SUNY Upstate Medical University, Syracuse, New York, NY, United States

**Keywords:** NMDA receptors, ketamine, D-serine, kynurenic acid, depression, schizophrenia, Alzheimer's disease, alcohol use disorder

N-methyl -D- aspartate receptors (NMDARs) have a complex role in the developing and mature brain. Disruptions in NMDAR signaling have been observed in different psychiatric disorders such as schizophrenia, depressive disorder, and Alzheimer's disease (AD) (1). The articles in this Research Topic further advance our knowledge on the complex role of NMDARs in normal and pathological conditions and explore the possibility of novel therapeutic uses of NMDAR modulators.

The NMDAR hypofunction hypothesis of schizophrenia (2) is the basis for the current use of NMDAR modulators in modeling of this disease in animals and as potential therapeutics.

In the review article, Pei et al. address the use of direct and indirect NMDAR glycine-site modulators, such as glycine, D-cycloserine, D-serine, glycine transporter 1 (GlyT1) inhibitors, and D-amino acid oxidase (DAAO) inhibitors in the treatment of clinical symptoms and cognitive impairments seen in schizophrenia. Reviewed preclinical and clinical studies suggest that indirect NMDAR glycine-site enhancers such as GlyT1 inhibitors (sarcosine) and DAAO inhibitors (sodium benzoate, TAK-831) seem to be more potent in clinical efficacy and with fewer side effects than direct NMDAR glycine-site agonists, including glycine, D-cycloserine, and D-serine.

Due to the fact that D-serine is one of the most frequently used NMDAR modulators and findings of its nephrotoxicity in rats, important is the review of Meftah et al. that summarizes current findings of the safety of D-serine treatment in different mammals, including humans. The toxicity of D-serine to endocrine, cardiovascular, gastrointestinal and extrapyramidal systems, with a special focus on the kidneys, is comprehensively discussed. The authors conclude that in humans D-serine appears to be safe at currently studied maximal doses and suggest that in future work even higher doses combined with DAAO inhibitors should be investigated.

The kynurenic acid (KYNA), an endogenous NMDA receptor antagonist, is elevated in the brain of patients with schizophrenia (3). Wright et al. utilized pre-natal exposure to kynurenine to model prenatal insult in rats and have found gender and circadian changes in the extracellular levels of glutamate, GABA and KYNA in rat hippocampi. The authors suggested that sex and time-dependent changes in hippocampal neuromodulation, elicited by prenatal KYNA elevation, may influence behavioral phenotypes, and have translational relevance to psychotic disorders.

Mallien et al. focused to identify the cellular substrates of psychosis induced by NMDAR hypofunction at post-adolescent stages. For these purposes, they have analyzed the effect of the inducible ablation of NMDARs in ErbB4 expressing cells, as neuregulin 1 and its receptor ErbB4 have been identified as schizophrenia-associated susceptibility factors that closely interact with NMDARs. They concluded that post-adolescent NMDAR deletion, even in a wider cell population than parvalbumin-positive interneurons, is not sufficient to generate behavioral changes that mimic psychiatric disorders.

With ketamine's demonstrated efficacy in the treatment of unipolar depression (4), there are emerging questions on the mechanism of actions underlying its observed fast clinical improvement and the potential role of NMDA transmission in bipolar depression. Yang et al. in their article highlight the importance of NMDAR transmission in the generation of mental representation during working memory. They further postulate that the very rapid, antidepressant effect of intranasal ketamine may involve the disruption of NMDAR-generated aversive mood states by the anterior and subgenual cingulate cortices, providing the opportunity for the return of top-down regulation by higher prefrontal cortex areas.

The effects of a single intravenous infusion of ketamine hydrochloride on magnetoencephalographic recordings in drug-free individuals with major depressive disorder performing an attentional task during scanning, have been investigated by Gilbert et al. Dynamic causal modeling was used to model effective connectivity of excitatory and inhibitory pathways. The authors provide additional support for the GABA disinhibition hypotheses of depression and the role of AMPA receptors in ketamine's antidepressant effects.

Dong et al. in their article address the impact of another oral NMDAR antagonist, D-cycloserine, combined with lurasidone on glutamate and glutamine in bipolar depression. This preliminary pilot study demonstrated that a lower mean glutamate level post-treatment after administration of NMDAR antagonist in combination with lurasidone predicts a better antidepressant response in bipolar depression. Authors propose that in the future, attenuation of the glutamate response to NMDAR antagonists could potentially be used as a biomarker for screening of NMDAR antagonists for their antidepressant potential.

Recent studies suggested that ketamine's rapid-acting antidepressant effect is potentially mediated by the opioid system (5). Bowman et al. have investigated the resting state electroencephalography profiles induced by co-administration of ketamine with either antipsychotic clozapine, or opioid receptor antagonist naltrexone, in freely moving rats to clarify this issue further. They demonstrated that the effect of clozapine, ketamine and naltrexone on local field potentials (LFP) depends of the locomotor state and that both clozapine and naltrexone modulated the effect of ketamine LFPs.

Balanced NMDAR activity is required for optimal brain and neurocognitive function (6). In an overview, Orzylowski et al. summarize the potential role of D-serine in normal and pathological aging such as AD. They review both preclinical and human studies of D-serine's modulation of cognition. Albeit controversial, it has been suggested that, in normal aging, decreased serine racemase expression, lower D-serine concentration, and NMDARs downregulation may lead to impaired synaptic plasticity and declined cognitive function. On the other hand, in AD, increased serine racemase expression, higher D-serine levels, and NMDAR overactivation tend to generate neurotoxicity and dementia. D-Serine and DAAO have been proposed as possible biomarkers and D-serine and DAAO inhibitors as potential therapeutics in early-phase AD.

Besides its role in schizophrenia and depression, the glutamatergic system and NMDARs have also been implicated in the pathophysiology of alcohol use disorder (7). Alcohol exposure upregulates Fyn, a protein tyrosine kinase that indirectly modulates NMDAR signaling by phosphorylating the NR2B subunit. Thompson et al. showed that saracatinib, the Src/Fyn kinase inhibitor, at the doses and regimen used in the study did not affect alcohol-seeking/craving or consumption in habitual mice or heavy drinking human participants.

## Author Contributions

All authors listed have made a substantial, direct, and intellectual contribution to the work and approved it for publication.

## Funding

This work was partially supported by National Health Research Institutes, Taiwan (NHRI- EX 111-11133NI), Ministry of Science and Technology, Taiwan (109-2314-B-039-039-MY3 and MOST 110-2622-B039-001), and China Medical University Hospital, Taiwan (DMR-111-243).

## Conflict of Interest

The authors declare that the research was conducted in the absence of any commercial or financial relationships that could be construed as a potential conflict of interest.

## Publisher's Note

All claims expressed in this article are solely those of the authors and do not necessarily represent those of their affiliated organizations, or those of the publisher, the editors and the reviewers. Any product that may be evaluated in this article, or claim that may be made by its manufacturer, is not guaranteed or endorsed by the publisher.
